# Valorization of Almond (*Prunus serotina*) by Obtaining Bioactive Compounds

**DOI:** 10.3389/fnut.2021.663953

**Published:** 2021-05-31

**Authors:** Claudia T. Gallardo-Rivera, Analía Lu, Mayra Z. Treviño-Garza, Eristeo García-Márquez, Carlos Amaya-Guerra, Carlos Aguilera, Juan G. Báez-González

**Affiliations:** ^1^Universidad Autónoma de Nuevo León, Facultad de Ciencias Biológicas, Departamento de Alimentos, Laboratorio de Reologia, San Nicolás de los Garza, Mexico; ^2^Centro de Investigación y Asistencia en Tecnología y Diseño del Estado de Jalisco, A.C., Autopista Mty-Aeropuerto Km 10 Parque PIIT, Apodaca, Mexico

**Keywords:** antioxidants, shell, paste, *Prunus serotina* oil, Capulin almond

## Abstract

The Capulin almond is a seed of the *Prunus serotina* (var. *capuli*) that belongs to the *Rosaceae* family. In this study, the valorization of the Capulin almond was performed by extracting antioxidants contained in the shell, paste, and oil (extracted by manual cold pressing process) of *Prunus serotina* treated with methanol, ethanol, acetone, and acidified water (pH 4) in a ratio of 1:5 (w/v). Total phenols were performed using the Folin-Ciocalteu method and expressed as gallic acid equivalents (GAE), antioxidant activity was determined by ABTS and DPPH methods and expressed as Trolox equivalents (TE). Finally, the total flavonoids were determined using a catechin calibration curve and reported as catechin equivalents (CE). The highest extraction of total phenols in shell was obtained with methanol (1.65 mg GAE/g sample) and the lowest using acidified water (0.97 mg GAE/g sample). However, extraction with acidified water favored this process in the paste (1.42 mg GAE/g sample), while the use of solvents did not influence it significantly (0.72 to 0.79 mg GAE/g sample). Regarding the total flavonoids, the values for the shell, paste, and oil were of 0.37, 0.78, and 0.34 mg CE/g sample, respectively, while that corresponding to the antioxidant activity evaluated with ABTS and DPPH were of 1527.78, 1229.17, 18894.44 μM TE/g, and, 568.45, 562.5 and 4369.05 mM TE/g sample, respectively. Finally, our results suggest that by-products such as the shell, paste, and oil obtained from *Prunus serotina* (var. *capuli*) represent a potential alternative for the recovery of bioactive compounds with antioxidant activity such as phenolic compounds and flavonoids.

## Introduction

The *Prunus serotina* tree, belonging to the *Rosace*a family, produces the Capulin fruit as a globose reddish-black drupe at maturity 12 to 20 mm in diameter, which contains an almond inside. This botanical variety is part of the four varieties (*alabamensis, capuli, rufula* and *serotina*) constituting the classification cited by Guzman et al. (1). Its geographical distribution includes southern Mexico, Guatemala, the United States and Canada (2). The *Prunus serotina* tree grows in mountainous regions at altitudes of 2,500 m or more in the Valley of Mexico and Guanajuato, as well as from Jalisco to Chiapas. In Mexico, the annual production of Capulin fruit was 184.87 tons, and it has been used since colonial times in the treatment of cardiovascular, respiratory and gastrointestinal diseases (3). Recent studies show that *Prunus* species may protect against metabolic syndrome, which includes sensitivity to insulin, visceral obesity, dysregulated metabolism of glucose and lipid, and hypertension. It can also be used to treat stress, immune problems, and anemia, as well as improve brain function (4–7). The Capulin fruit is consumed fresh and dehydrated, as snack and ingredient in processed foods such as jellies, jams and liqueurs (8, 9). The Capulin almond is comprised of three parts: the edible part of the almond (kernel), which has a thin shell (skin) that wraps around the kernel, the skin, and an external part called the shell. Various studies have shown that almond by-products (of the kernel, skin, and shell) contain bioactive compounds such as phenolic compounds (flavonoids and phenolic acids) and terpenoids (sterols and triterpenoids), whose composition and quantity depend on factors such as the geographical distribution, origin, environmental conditions, exposure to pests, UV radiation, harvest maturity and obtaining and extraction process (10–14). These by-products are a source of potent antioxidants for the control of oxidative processes, natural antimicrobials, prebiotic and antiviral compounds (15–18).

The almond of *Prunus serotina* is a source of lipids, raw fiber, humidity, and carbohydrates, in addition to containing vitamin E and minerals such as Ca, Fe, Mg, P, K, Zn, and Na (8). The Capulin almond stands out for its high level of ∝-eleostearic acid (27%), which is effective in the suppression of the growth of cancer cells and possesses antihypertensive properties due to the presence of vasodilator compounds, such as urosolic acid and uvol. It is also effective as an antiparasitic and antimicrobial (4, 6, 19). Tests on the oil extracted from *Prunus serotina* seeds (with hexane and supercritical CO_2_), have shown that the oil was highly polyunsaturated with predominant content of oleic acid (35%), followed by ∝-eleostearic (27%), linoleic (27%), palmitic (4%), stearic (4%), and β-eleostearic (1%) acids (20). Studies in *Prunus amygdalus* Batsch ssp dulce in the kernel of the almond indicate a range in oil content of 36.76 to 79%, depending on the genotype and a total content of polyphenols between 23.75 and 98.67 mg GAE/100 g, with and antioxidant activity between 44.59 and 91.18% using DPPH (1,1-diphenyl-2-picrylhidrazyl). Likewise, the presence of K, P, and Ca as predominant minerals was determined (16).

The analyzes in *Prunus dulcis* Mill D.A. Webb show that the kernel of the ripe almond contains around 50% lipids, 25% protein and ~20% carbohydrates. It has a low moisture content and various bioactive compounds in small amounts. The polyphenols are some of these compounds that are related to the quality of the almond and help to increase its shelf-life. Regardless of the type of almond, it has been reported that polyphenols have been seen in a range of 61 to 162 mg/100 g. However, flavonoids (87 to 135 mg/100 g) are by far the most abundant compounds in almonds. Some studies indicate that the quality of the kernel of the almond depends on the harvest time, increases in the lipid content and decreases in the carbohydrate and protein content. Late harvest may increase antioxidant activity, suggesting that antioxidant compounds develop late in ripening (19). Additionally, almond, walnut, and pine nut shells have been studied by recovering their bioactive compounds. It was found that the ethanol-water extracts contained total polyphenols, flavonoids, condensed tannins and antioxidant activity values of IC_50_ 7.9, 15.2 and 8.2 μg/L, respectively. Moreover, the chemical composition and structure of the shell confers brittleness and fracture behavior. The chemical differences between these are also found in the content of polysaccharides, hemicellulose profile and extractives. The foregoing suggests a potential for the reuse mainly of polysaccharides (hemicellulose and lignin) (21). The polyphenols extracted from the *Prunus dulcis* almond have shown their potential use as a natural dietary antioxidant whose effect depends on its composition and bioavailability. Its content in this almond is comparable to nutrients such as lipids and fiber (8).

The kernel of this almond stands out for its nutritional and commercial value. However, its by-products such as skin, shell and hull are distinguished by their content of phenolic acids and flavonoids that can be used with applications in food and cosmetic formulations. The skin constitutes 4% of the total weight of the almond but contains between 60 and 80% of the total phenolic compounds present in the seed. Regarding the almond shell, it is composed of cellulose (29.8 to 50.7%), hemicellulose (19.3 to 29%) and lignin (20.4 to 50.7%). The almond shell constitutes 35 to 62% of the total weight of the fresh almond, whose weight differs significantly due to variety and agronomic factors. It is peel has been reported to contain 18 to 30% sugar, 2.1 to 8.8% protein, 10 to 24.9% crude fiber, cellulose from 20.6 to 35.2%, and crude lignin in a range of 7.5 to 15.6% (5, 12, 22).

Various studies report the total content of phenolic compounds in the kernel (8±1 mg QCE/g ethanolic extract to 8.1±1.75 mg CE/g ethanolic extract), skin (87.8±1.75 mg CE/g ethanolic extract to 88±2 mg QCE/g ethanolic extract), shell (38±3.30 mg GAE/g methanolic extract) and hull (71.1±1.74 mg CE/g ethanolic extract to 78.2±3.41 mg GAE/g methanolic extract) of the almond (*Prunus amygdalus* L.) (9, 23, 24). These antecedents show the viability in the valorization of various types of *Prunus* almond focused on the extraction of the oil from the skin and shell. The objective of this study was to value the by-products (shell, paste and oil) of the *Prunus serotina* almond through the manual cold pressing process (30°C). Considering the content of phenolic compounds and flavonoids in these by-products, the *Prunus serotina* almond is an alternative for the recovery of more valuable by-products than that of the *Prunus dulcis* almond, which is the most studied seed.

## Materials and Methods

The Capulin almond was purchased in a local market in Mexico City. We used analytical solvents such as ethanol, methanol, acetone, hexane, boric acid, phosphoric acid, hydrochloric acid, butanol, glacial acetic acid, isooctane, propanol, boron trifluoride, heptane, potassium persulfate, ammonium thiocyanate, chloride barium, and sulfate ferrous anhydrous (JT Baker reagents, Mexico). Organic compounds such as Folin and Ciocalteu, gallic acid, catechin hydrate, (2,2′-azinobis 3-ethylbenzohtiazoline-6 sulfonic acid) ABTS, DPPH, Trolox, 1,1,3,3-Tetraethoxypropane, thiobarbituric acid acquired from Sigma-Aldrich (Sigma-Aldrich, Mexico) were also utilized.

### Extraction Oil

**Figure 1:**
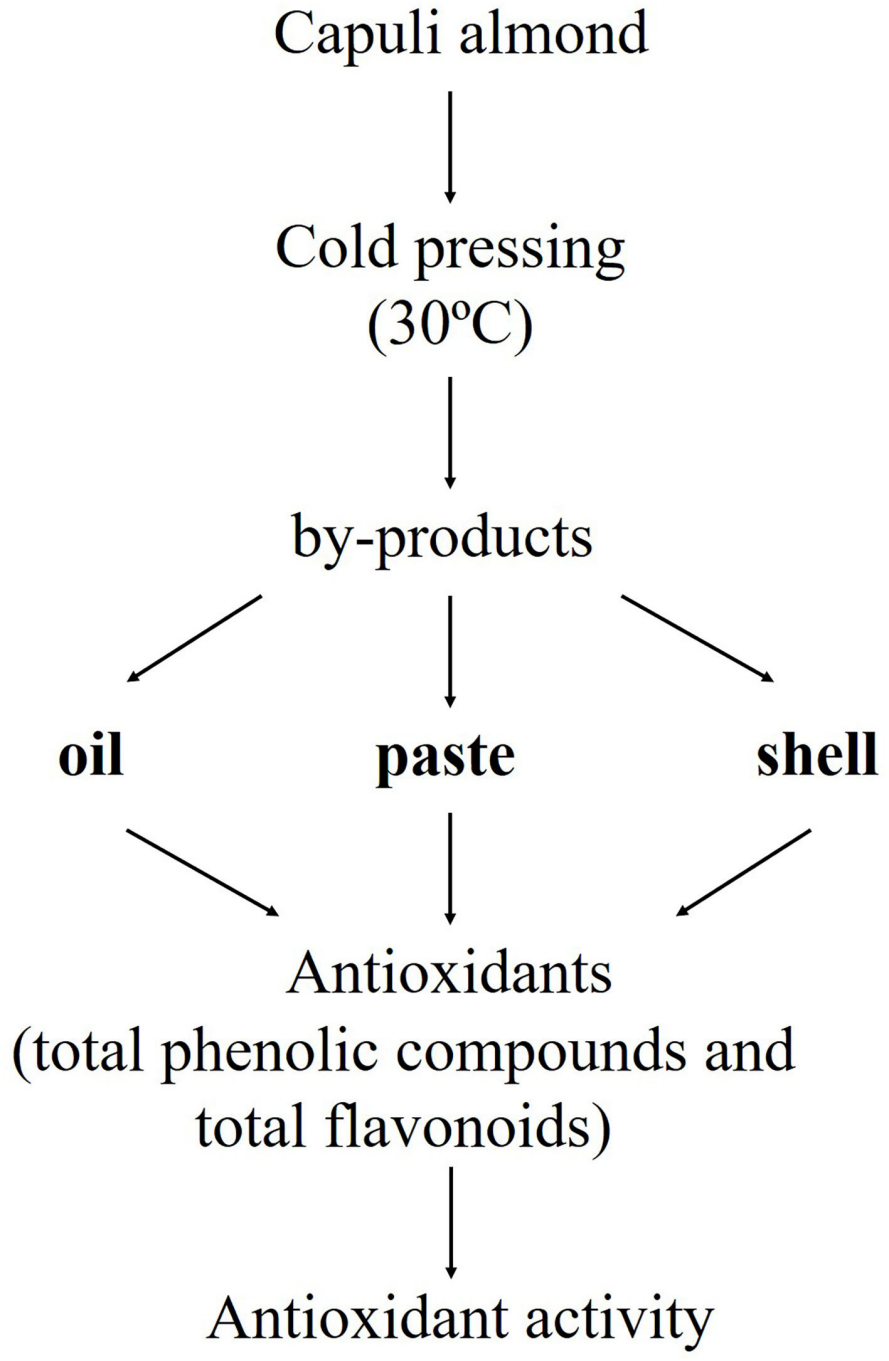
Process of valorization of the *Prunus serotina* (var. *capuli*) almond.

**Figure 2:**
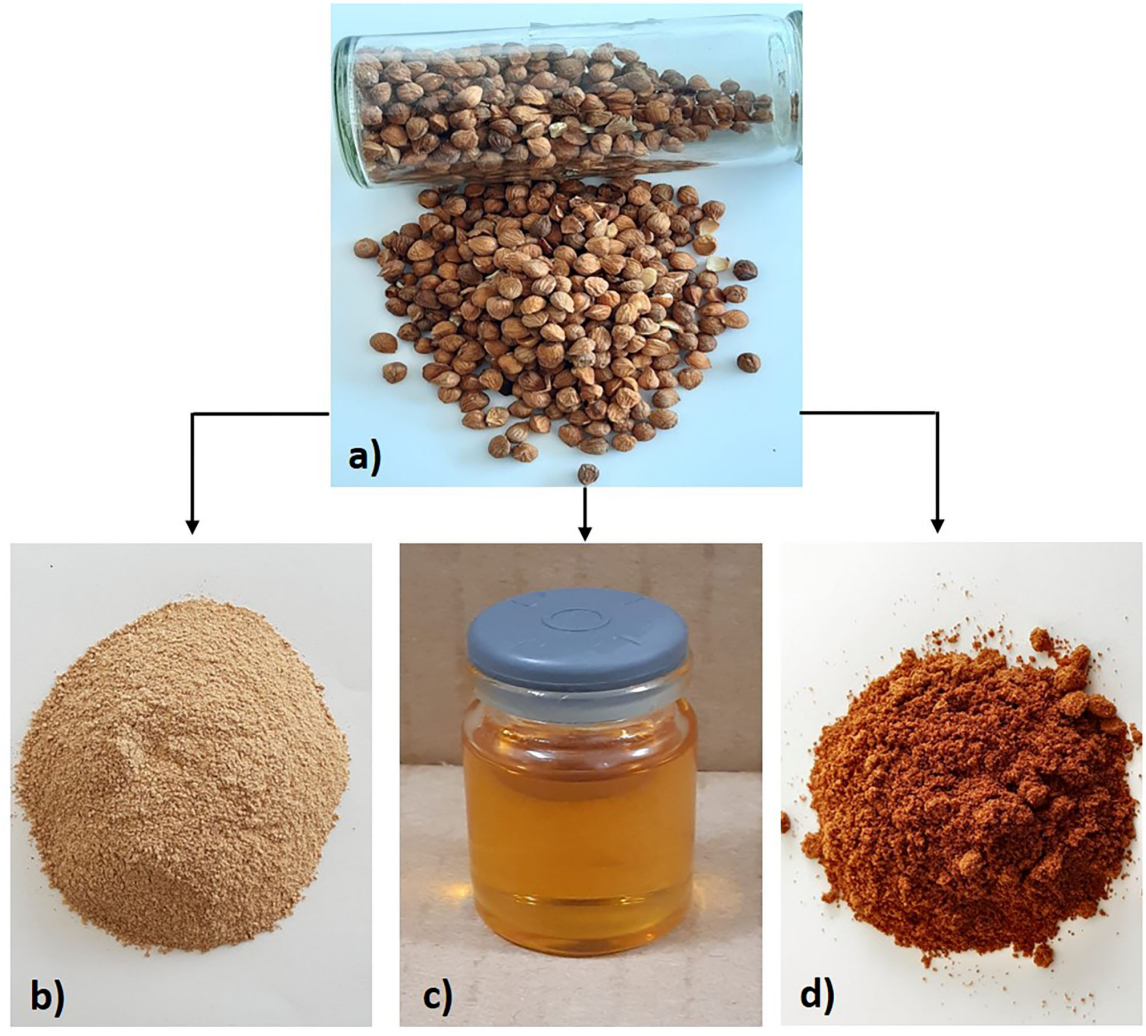
**(a)** *P. serotina* (var. capuli) seed and their by-products; **(b)** shell, **(c)** oil and, **(d)** paste.

The seed of the Capulin almond was obtained by a cracking process (as seen in Figure 1) using a sterilized metal squeezer and was stored in airtight bags at −20°C until used. The oil was extracted from seed (100 g) by manual cold pressing (Henan Wecare Industry Co. Ltd, China) at a temperature of 30°C; the oil and paste of *Prunus serotina* by-products were stored in amber jars and hermetic bags at −20°C (25) (Figure 2) until later use.

### Preparation of Samples of Almond By-Products

The extraction of antioxidants compounds in by-products of *Prunus serotina* such as oil (manual cold pressed), paste (kernel + skin), and shell was carried out with the procedure of Pinelo et al. (26). We mixed 1 g of oil with a solvent (such as ethanol, methanol, acetone (all at 96%) and acidified distilled water (pH4) for 4 min in a vortex, after which the suspension was separated using a centrifuge at 3,000 rpm for 5 min (LABORTECHNK, Wehingen, Germany) (27). The paste and shell were ground individually in a food processor (NUTRIBULLET, China) (Figure 2), each sample being mixed with solvent (w/v) in a 1:5 ratio and placed in a water bath (50°C) with magnetic stirring for 90 min. The solids were separated by centrifugation at 800 rpm for 5 min and the supernatant collected for further analysis (28).

### Total Phenolic Compounds

The total phenolic compounds extracted with the different solvents were quantified according to the Singleton et al. (29) procedure with some modifications. The sample of supernatant 1,000 μL obtained from oil, paste, and shell mixed with 100 μL of Folin and Ciocalteu reagent 1N was allowed to stand for ~5 min. Then 300 μL of sodium carbonate 20% was added and adjusted to a volume of 2,700 μL with distiller water, the mixture was kept a rest for 90 min in dark condiction. We measure the absorbance of the sample with a previous rest of 90 min at 765 nm using a UV spectrophotometer in dark condition at 25 ± 2°C (Thermo Fisher Scientific Equipment, MA, USA). The calibration curve was obtained with Gallic acid in a concentration range of 0 to 53 mg/L. The content of phenolic compounds was reported as the mg gallic acid equivalents/g of extract (mg GAE/g) (30). Finally, in order to homologate the use of the solvent in the extraction process of the bioactive compounds from almond by-products, we decided to use ethanol as solvent in the subsequent evaluations since it showed a good yield and content of total phenols (26). The solvent yield was determined according to the following equation:

$$\begin{array}{l} Yield\;\left(\%\right)=\;\frac{Final\;volume\;of\;solvent\;}{Initial\;volume\;of\;solvent}\;x100 \end{array}$$

### Total Flavonoids

Flavonoid compounds were analyzed by the colorimetric method proposed by Esfahlan et al. (9) with slight modifications. We mixed 250 μL of sample extract (shell, oil and paste) with 1,225 μL of distilled water contained in a glass cell, 75 μL of NaNO_2_ 5%, 150 μL of AlCl_3_ 10% and 500 μL NaOH (1M) added with a time difference of 5 min, respectively. We measured the absorbance with a spectrophotometer (Thermo Fisher Scientific, MA, USA) at 510 nm before 30 min. The standard curve made using catechins in an range of 1.6 to 16 mg/L. The results of the triplicate test were expressed for each by-product as catechin equivalents/g of extract (mg CE/g).

### Antioxidant Activity

#### Method ABTS (2,2′-Azino-bis (3-Ethylbenzothiazoline-6-Sulphonic Acid)

The antioxidant activity was evaluated using the Fernandes et al. (31) method with some modifications. The radical ABTS was prepared by mixing potassium persulfate (2.45 mM) and ABTS (7 mM) in a 1:1 ratio (v/v), and the solution stored in a dark environment for 16 h. 200 μL of radical ABTS diluted with 10 ml of ethanol (96 %). The resulting solution showed an absorbance of 0.700 ± 0.05 absorbance at 734 nm in a UV spectrophotometer (Thermo Fisher Scientific, MA, USA). The standard curve was generated using Trolox in a concentration interval of 25 to 505 μmol. After adjusting the optical density, the 100 μL of the extract prepared with 2,000 μL of ABTS radical (by triplicate). This mixture was homogenized and analyzed at 734 nm (then 7 min applying the mixture). The results were expressed as μmol equivalent of Trolox/g sample (μM TE/g sample) (32). In addition, the ABTS radical scavenging activity was determined according to the following equation:

$$\begin{array}{l} Inhibition\;\left(\%\right)\\ =\frac{Absorbance\;of\;ABTS\;solution-Absorbance\;of\;sample\;}{Absorbance\;of\;ABTS\;solution}\;x\;100 \end{array}$$

### Method DPPH (2,2-Diphenyl-1-Picrylhydrazyl)

The antioxidant activity was evaluated according to the Moosavi et al. (33) method to corroborate the behavior obtained by the ABTS method and inhibition of radicals from phenolic compounds. In this method, the DPPH (2,2-diphenyl-1-picrilhydrazyl) radical generated by the addition of 96% ethanol to a 0.1 mM solution of DPPH until absorbance values reached 1 to 571 nm was measured with UV spectrophotometer (Thermo Fisher Scientific, MA, USA). The standard curve was obtained with Trolox in a concentration range of 25 to 505 μmol. We mixed 50 μL of the sample extract and 2,000 μL of the DPPH radical, and the mixture remained in the dark, for 30 min at 25 ± 2°C to measure the absorbance at 517 nm. The results of the triplicate analysis were expressed as μmol equivalent of Trolox/g sample (μM TE/g sample) (32). In addition, the DPPH radical scavenging activity was determined according to the following equation:

$$\begin{array}{l} Inhibition\;\left(\%\right)\\ =\frac{Absorbance\;of\;DPPH\;solution-Absorbance\;of\;sample\;}{Absorbance\;of\;DPPH\;solution}x100 \end{array}$$

### Statistical Analysis

Data analysis was performed by calculating the mean value and standard deviation of the triplicate determinations to evaluate their repeatability. It was determined that there is a significant difference between the means (at p ≤ 0.05) through a completely random analysis of variance (ANOVA). Finally, the multiple comparison of significant means was evaluated using the Tukey test.

## Results

### Solvent Yields and Total Phenol Extraction

**Figure 3:**
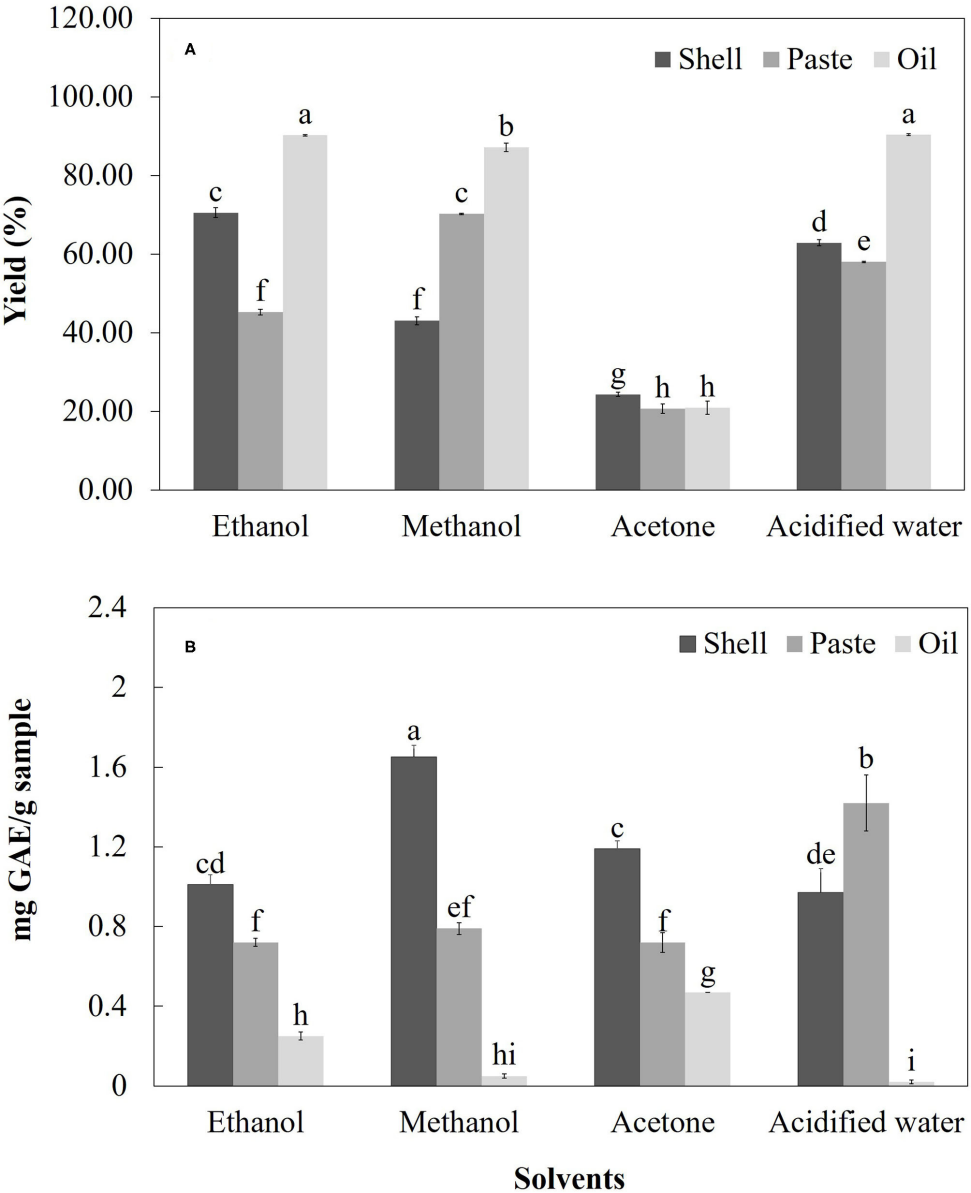
**(A)** Yields of different solvents and **(B)** extraction of total phenolics of various almond by-products. The vertical bars indicate mean ± standard deviation (*n* = 3).

Figure 3 shows the yields of various solvents (ethanol, methanol, acetone and, acidified water) used to obtain total phenolic compounds the almond shell, oil (cold press) as well as the paste (kernel + skin) resulting from the oil extraction process. Significant differences (*p* > 0.05) were found both in the yield of the solvents and in the extraction of the phenolic compounds. In the case of the shell, ethanol presented the highest yield as a solvent (70.53%; 1.01 ± 0.05 mg GAE/g). However, methanol was the most effective for the extraction of total phenolic compounds (43.07%; 1.65 ± 0.06 mg GAE/g). In addition, the acidified water presented a solvent yield of 62.87%, with a total phenol content of 0.97 ± 0.12 mg GAE/g. On the other hand, the lowest yield was for acetone with values of 24.33% and 1.19 ± 0.04 mg GAE/g. Regarding paste, the highest solvent yield was for methanol (70.20%; 0.79 ± 0.03 mg GAE/g), yet the total phenolic content was higher in the extraction with acidified water (58.00%; 1.42 ± 0.14 mg GAE/g). In addition, the total phenolic content was similar with the extractions with ethanol (45.20%; 0.72 ± 0.02 mg GAE/g) and acetone (20.67%; 0.72 ± 0.05 mg GAE/g), respectively (Figure 3). Moreover, in the case of oil, although the solvent yields were higher for acidified water (90.33%; 0.02 ± 0.01 mg GAE/g) and ethanol (90.20%; 0.25 ± 0.02 mg GAE/g), the total phenolic content was higher in the acetone extraction (20.87%; 0.47 ± 0.00 mg GAE/g). Finally, the yield in the extraction with methanol was 87.13% and the total phenolic content was 0.05 ± 0.01 mg GAE/g (Figure 3). In general, it can be observed that the use of different solvents significantly influenced the obtaining of total phenolic compounds from the various by-products.

### Flavonoid Content Determination

**Figure 4:**
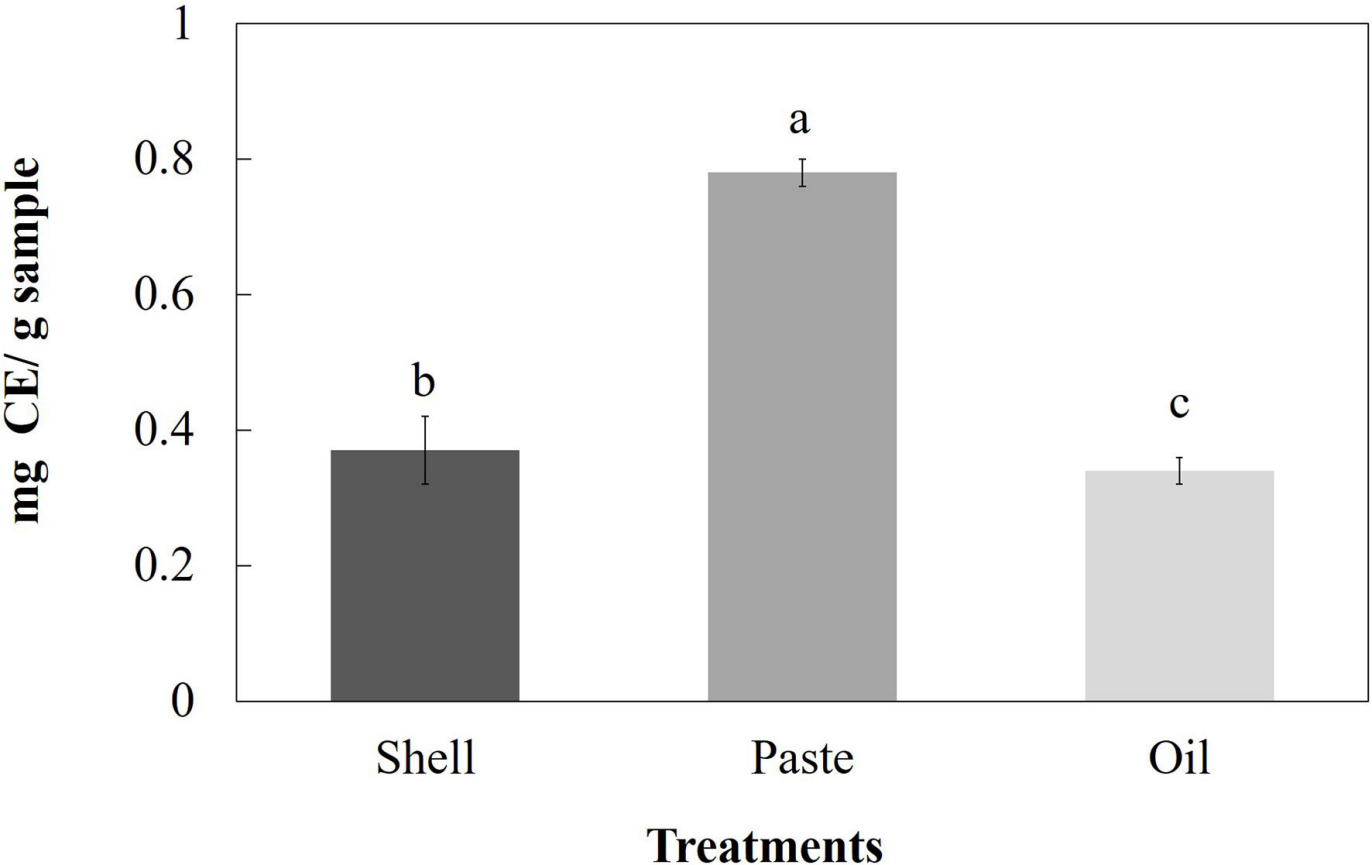
Flavonoid content of various almond by-products extracted with ethanol. The vertical bars indicate mean ± standard deviation (*n* = 3).

As shown in Figure 4, a significant difference (*p* > 0.05) was found regarding the content of total flavonoids in the various by-products of the almond. The highest values were found in the paste, and the lowest values in the oil (0.78 ± 0.02 mg and 0.34 ± 0.02 mg EC/g, respectively). In the case of shell, the content of total flavonoids was 0.37 ± 0.05 mg EC/g.

### Antioxidant Activity Determination

**Figure 5:**
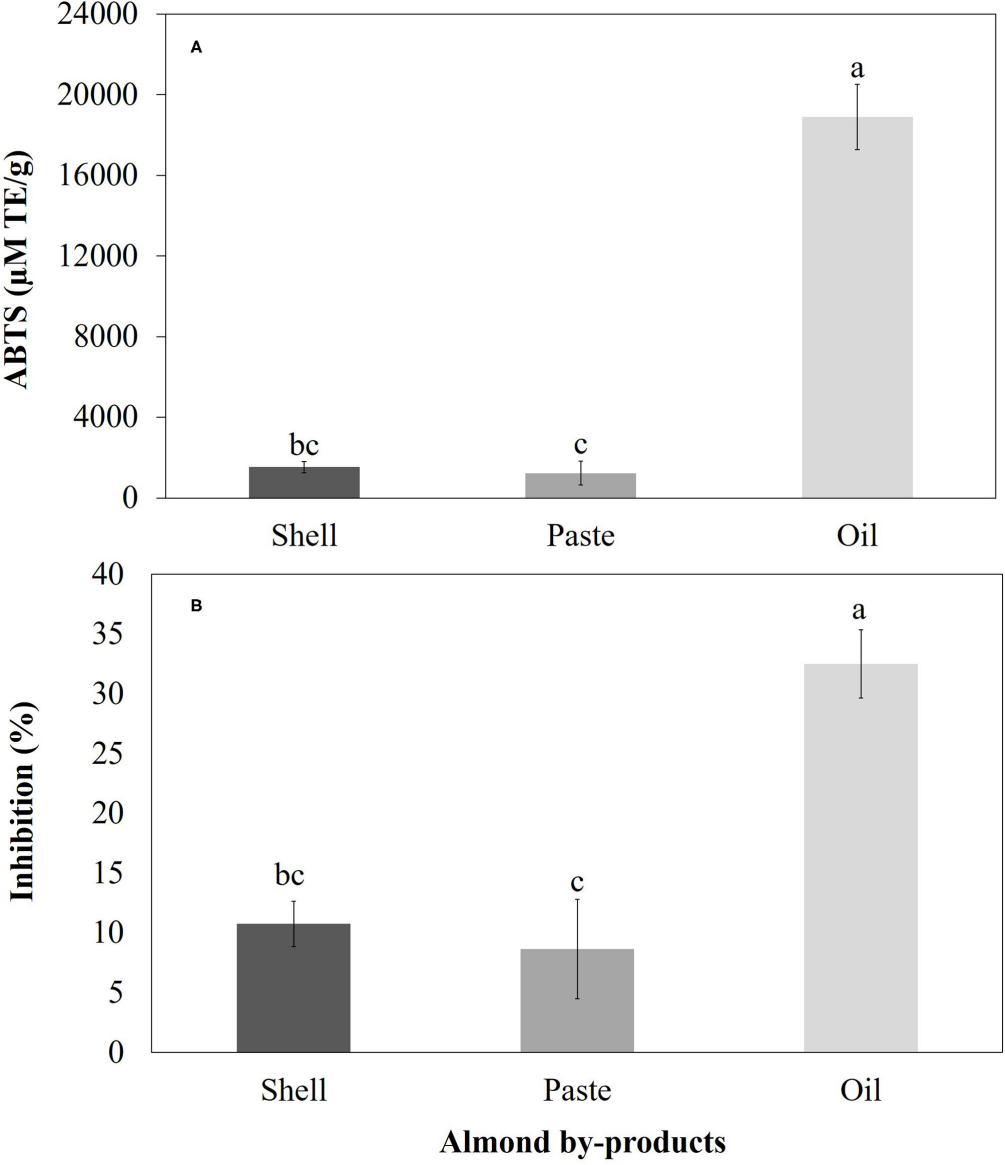
**(A)** Antioxidant activity and **(B)** ABTS radical scavenging activity of various almond by-products extracted with ethanol. The vertical bars indicate mean ± standard deviation (*n* = 3).

**Figure 6:**
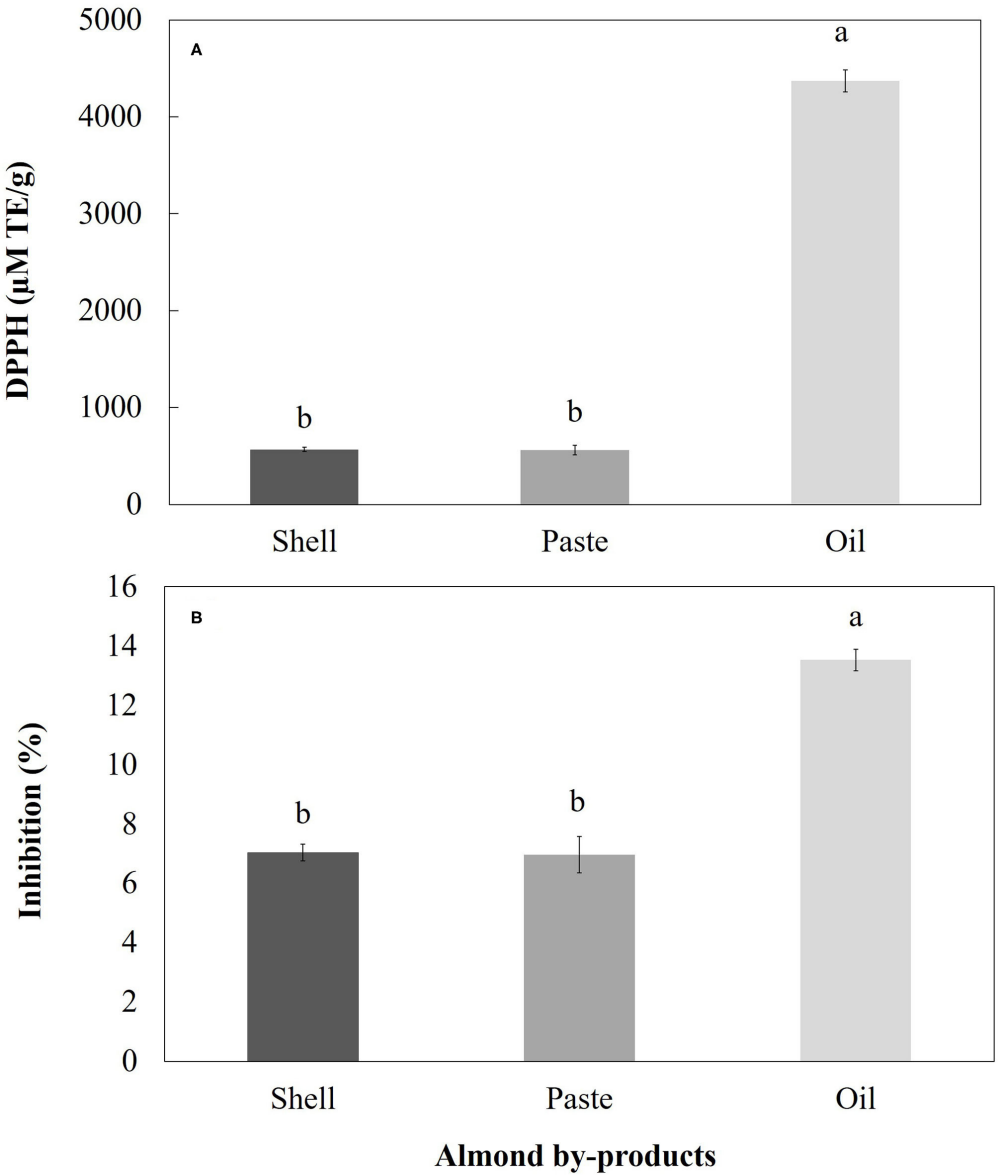
**(A)** Antioxidant activity and **(B)** DPPH radical scavenging activity of various almond by-products extracted with ethanol. The vertical bars indicate mean ± standard deviation (*n* = 3).

Regarding the antioxidant activity measured by the DPPH and ABTS methods, a significant difference (*p* > 0.05) was found between the different treatments. In both methods, the highest values were for oil, followed by shell and paste. The antioxidant activity values found in the shell were 1,527.78 ± 268.69 78 μM TE/g (% inhibition = 10.77 ± 1.89) and 568.45 ± 22.47 μM TE/g (% inhibition = 7.04 ± 0.28) for ABTS and DPPH, respectively (Figures 5, 6). On the other hand, the antioxidant activity values of the pasta were 1,229.17 ± 587.78 μM TE/g with a % inhibition of 8.66 ± 4.14 (ABTS) and 562.50 ± 49.71 μM TE/g with a % inhibition of 6.97 ± 0.62 (DPPH) (Figures 5, 6). Finally, in the case of oil, the antioxidant activity values obtained in this research were 18,894.44 ± 1,625.18 μM TE/g with a % inhibition of 32.50 ± 2.86 and 4,369.05± 114.81 μM TE/g with a % inhibition of 13.532 ± 0.36 (Figures 5, 6).

## Discussion

### Solvent Yields and Extraction of Total Phenolics

In the case of the shell, the results found in terms of solvent yield indicated that, although ethanol presented the highest yields, methanol was the most effective solvent for the extraction of total phenolic compounds (Figure 3); this effect is in line with that reported by Pinelo et al. (26) during the extraction of antioxidant phenolics from almond hulls. Moreover, although the acidified water presented a good yield as a solvent, it also showed the lowest value in the total phenol content; this finding is similar to that reported by Jiménez et al. (34). Moreover, although the acetone showed a total phenol content similar to that obtained by ethanol, it also showed the lowest yield (Figure 3); this effect could be related to the high volatility of the acetone with respect to other solvents. Finally, the values of the total phenol content of the almond shell found in this research are lower than those reported in *P. dulcis* (21, 22, 35, 36), *P. amygdalus* (33, 37), and *P. serotina* Ehrh (7). However, they are similar to those reported by Moure et al. (24) in *P. amygdalus* and Bolling et al. (38) in California almonds. In the case of paste, although the yield was higher for methanol, the total phenolic content was higher in the extraction with acidified water (Figure 3). Compared with previous studies, the content of total phenols found in this study was lower than that reported in various commercial *P. dulcis* cultivars (39, 40) and higher than that reported in various genotypes of *P. amygdalus* almond (16). In the case of oil, although the highest yields were found in ethanol and acidified water, the highest total phenol content was found in acetone as a solvent (Figure 3). Compared with other studies, the content of total phenols of the oil obtained in this research was lower than that reported by Mezzomo et al. (41) in peach (*P. persica*) almond oil and higher than that reported by Miraliakbari (42) in *P. dulcis* oil.

In general, the highest content of total phenols was found in the shell, followed by the paste and the oil. These findings are in agreement with those reported by Isfahlan et al. (37) in Iranian almond, indicating that the outer layers of the almond, such as the shell, contain a greater amount of phenolic compounds linked to the components of the cell wall, such as proteins and polysaccharides, and their purpose is to protect internal materials. Likewise, Luna-Vázquez et al. (7) report a higher content of total phenolic compounds in the peel of Black Cherry *P. serotina* compared to the flesh. In addition, the differences in our results with respect to that reported in other research could be associated with the diversity of the almond species (43), the cultivars, the growing regions, and environmental factors, as well as the product's harvest season, among others (11, 39). Furthermore, it is important to mention that the total phenol content is also influenced by the solvent used in the extraction process (2, 26, 37). Finally, in order to homologate the use of the solvent in the extraction process of the bioactive compounds from almond by-products, we decided to use ethanol as solvent in the subsequent evaluations since it showed a good yield and total phenol (26, 34).

### Flavonoid Content Analysis

Flavonoids are a group of phenolic compounds that includes anthocyanidins, isoflavones, flavanones, flavonols, and flavanols, among others (19). These compounds are associated with beneficial effects due to their wide variety of antioxidant properties (they act as scavengers of free radicals), antimicrobial, anti-inflammatory, anticancer, vasodilatory, among others (33, 39). As mentioned in the Results section, the total flavonoid content was higher in the almond paste, followed by the shell and oil (Figure 4). In the case of pasta, the content of total flavonoids found in this research (Figure 4) was higher than that reported in *P. serotina* Ehrh (7), but lower than that reported in *P. dulcis* (39, 40), *A. pabotti* and *A. korschinskii* (43). On the other hand, in the case of the shell, our results (Figure 4) showed a lower content of flavonoids than that reported in *P. dulcis* (21), but a higher content compared to that reported in *P. serotina* Ehrh (7); This effect may be associated with the variety of the almond and with the seasonal harvest of the product (38). Finally, the total flavonoid values found in the oil (Figure 4) were higher than those reported by Karaman et al. (44) in different cold press edible oil by-products (almond, walnut, pomegranate, and grape), but very similar to that reported in a previous study conducted by our research group (25). In general, most of the flavonoids in almonds are present in the external part of the fruit, acting almost exclusively on the skin and protecting the seed against environmental stress (37).

### Antioxidant Activity Evaluations

The antioxidant activity of almonds is attributed to its content of phenolic compounds in various parts of the fruit, such as the hull, shell, skin and, kernel, whose main components are phenolic acids (e.g., non-flavonoids such as benzoic acids and cinnamic acids), and flavonoids (e.g., flavanols, flavonols, flavanones and procyanidins) (12). As shown in the Results section, regarding the antioxidant activity measured by the DPPH and ABTS methods, the highest values were found in the oil, followed by the shell and paste (Figures 5, 6, respectively). The antioxidant activity values found in the shell are lower than those reported in *P. serotina* Ehrh by Luna-Vázquez et al. (7) but similar to those reported in some genotypes (S1-1, S4-3 and S4- 6) of *P. amygdalus* (33), and *P. dulcis* (21). On the other hand, the antioxidant activity values found in pasta (Figures 5, 6) are lower than those reported in *P. serotina* Ehrh. (7) and *P. amygdalus* L. (16, 43), but higher than those reported by Summo et al. (11) in *P. dulcis*. Finally, in the case of oil, the antioxidant activity values obtained in this research (Figures 5, 6) were similar to those reported in *P. serotina* by Lu-Martínez et al. (25) and higher than those reported in *P. dulcis* by Miraliakbari (42). Finally, as mentioned in the previous sections, the fluctuations in terms of the results found in this research and the studies reported in the literature can be attributed to the diversity of species and varieties within almond species (7).

In general, although the content of phenolic compounds was higher in the shell, the pasta showed the highest content of flavonoids. In addition, the highest antioxidant activity was found in the oil; this effect may be associated with the content of phenolic compounds and to the presence of non-phenolic compounds with antioxidant activity such as terpenoids (sterols and triterpenoids) (12).

## Conclusions

It was possible to obtain phenolic compounds from almond by-products, such as shell, paste, and oil using various solvents. In general, it can be observed that the use of different solvents significantly influenced the obtaining of total phenolic compounds from the various by-products. In the shell, ethanol showed the highest yields, but methanol was the most effective solvent for obtaining the total phenolic compounds. Regarding paste, although the yield was higher for methanol, the total phenolic content was higher in the extraction with acidified water. In addition, ethanol and acidified water showed the highest yields in the case of oil, but acetone was the most effective solvent for obtaining total phenolic compounds. In general, the highest content of total phenols was found in the shell, followed by the paste and the oil. Particularly, regarding total flavonoid content extracted with ethanol, the highest values were found in the paste, followed by the shell and oil. Moreover, regarding antioxidant activity obtained by ABTS and DPPH methods, the highest values were found in the oil, followed by the shell and paste. Finally, our results suggest that by-products such as the shell, paste, and oil obtained from *Prunus serotina* var capuli represent a potential alternative for the recovery of bioactive compounds with antioxidant activity, such as phenolic compounds and flavonoids. However, as prospects, it is recommended to continue with this research to determine various factors affecting the extraction of bioactive compounds and characterize the phytochemical compounds (e.g., polyphenols and flavonoids) found in the shell, oil and, paste of the Capuli almond, since these compounds are of interest due to their wide variety of properties associated with beneficial health effects.

## Data Availability Statement

The raw data supporting the conclusions of this article will be made available by the authors, without undue reservation.

## Author Contributions

JB-G, EG-M, MT-G, and CG-R: conceptualization. AL, MT-G, EGM, JB-G, CA-G, CA, and CG-R: investigation. CG-R, MT-G, and AL: writing—original draft preparation. MT-G, CG-R, EG-M, and JB-G: writing—review and editing. All authors contributed to the article and approved the submitted version.

## Conflict of Interest

The authors declare that the research was conducted in the absence of any commercial or financial relationships that could be construed as a potential conflict of interest.
